# The CRIP effect: How a pattern in central vision interferes with perception of a pattern in the periphery

**DOI:** 10.1167/jov.25.2.10

**Published:** 2025-02-26

**Authors:** Carolina Maria Oletto, Giulio Contemori, Esma Dilara Yavuz, Luca Battaglini, Michael Herzog, Marco Bertamini

**Affiliations:** 1 Department of General Psychology, University of Padova, Padova, Italy; 2 Laboratory of Psychophysics, Brain Mind Institute, École Polytechnique Fédérale de Lausanne, Lausanne, Switzerland

**Keywords:** peripheral vision, central vision, iso-orientation suppression, pattern perception, crowding

## Abstract

Our percept of the world is the result of interactions between central and peripheral vision. They can be facilitatory, because central vision is informative about what is in the periphery, or detrimental, such as when shape elements are pooled. We introduce a novel phenomenon, in which elements in the central region impair perception in the periphery (central region interference with periphery [CRIP]). We showed participants a squared grid containing small lines (vertical or diagonal) or crosses in the central region and diagonal lines in the periphery. The regions were divided by a gap that varied in size and position. Participants reported the orientation of the diagonal lines in the periphery (/ or \). The central pattern caused interference and hindered discrimination. For a fixed eccentricity of the peripheral elements, the smaller the gap the larger the impairment. The effect was only present when the central and peripheral lines had a shared orientation (i.e., diagonal), suggesting that similarity plays a role. Surprisingly, performance was worse if central and peripheral lines had the same orientation. We conclude that people do not rely on extrapolation when perceiving elements in the periphery and that iso-orientation may cause greater interference.

## Introduction

Peripheral resolution is much lower than resolution in the fovea. Still, we usually do not experience peripheral vision as poor. This phenomenon has been defined the “grand illusion” (Blackmore, Brelstaff, Nelson, & Trościanko, 1995; Dennett, 1993; Noë, 2002). For example, under normal conditions, a face in the periphery is perceived as the same face when foveated. Only when we covertly attend to the periphery do we see that the face appears as blurry and distorted, particularly when it is presented together with other objects or faces, a phenomenon referred to as crowding. Why peripheral vision appears as normal under normal conditions is largely unknown. A set of hypotheses claims that peripheral vision is extrapolated from central vision, in particular when uniform structures prevail; for example, identical patterns present in both central and peripheral vision may reinforce each other.

Here, we study a type of stimulus configuration in which a pattern with line elements extends over the central region, and a similar pattern extends in the periphery. We report a phenomenon that we refer to as central region interference with periphery (CRIP). The CRIP effect occurs when peripheral elements are perceived but their features are not correctly discriminated. The closer the central region to the outside region, the greater the interference. This effect is related to crowding, and a comparison is included in the final discussion. Before introducing the experiments, we briefly review the literature in which the CRIP effect is placed.

The foveal feedback hypothesis claims that central vision is involved through feedback in processing shape even for stimuli presented in the periphery (Contemori, Oletto, Battaglini, & Bertamini, 2024; Oletto, Contemori, Bertamini, & Battaglini, 2022; Williams et al., 2008). Scene information is correlated across different locations; therefore, information from central vision may be used to infer the overall scene. One of the possible mechanisms is the extrapolation of information from the fovea to the periphery (Suzuki, Wolfe, Horowitz, & Noguchi, 2013). Extrapolation is known to occur for surface brightness (Toscani, Gegenfurtner, & Valsecchi, 2017), motion (Wu, Kanai, & Shimojo, 2004), and even facial emotions (Zoghlami & Toscani, 2023). In general, the idea of extrapolation fits nicely in the broader area of predictive processing (Clark, 2013; Friston, 2009). Because information in the periphery is more uncertain than in the fovea, in uncertain cases information from the fovea may take over, which would be the prior in predictive coding terminology. Classic examples are filling in of information in the blind spot or healing grid illusion (http://www.illusionoftheyear.com/2005/healing-grid/) and the uniformity illusion.

**Figure 1.**
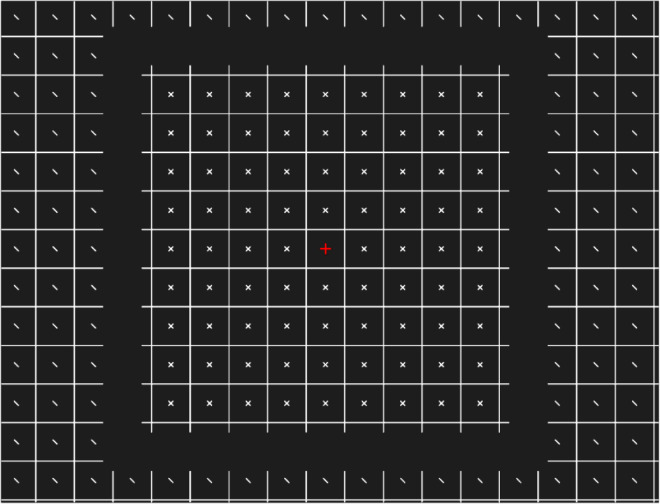
Example of a stimulus presented in the study. The image is cropped and the contrast is increased for a better resolution. In the real experiments, the contrast was lower and the peripheral elements extended farther into the periphery. Better resolution figures can be found at https://osf.io/sy64w/.

Here, we present a new effect, the CRIP effect, and show that foveal information does not lead to extrapolation but it is rather the other way around. Our stimuli consist of extended patterns separated by a gap. We created a non-uniform stimulus in which central and peripheral elements differ. The pattern was a squared grid with small additional lines, as shown in Figure 1. The lines in the periphery (targets) were always tilted ±45° with respect to the central axis, whereas in the center they varied depending on the experiment (distractors). By inserting a gap that divided central and peripheral elements, we tested how central elements influence (a) phenomenal appearance, and (b) discrimination of peripheral orientation. We were particularly interested in the effect of type of distractors and gap size.

In Experiment 1, we demonstrated the existence of the CRIP effect. In the other three experiments we manipulated the shape and luminance (Experiment 2) and orientation of the central lines (Experiments 3 and 4). The aim was to understand which characteristics of the distractors were causing the interference. In Experiment 5, we controlled for the effect of the transient onset of the stimuli by placing a grid between stimuli presentations. We also investigated whether participants could detect the shape of the elements in the periphery—that is, whether they perceived the lines despite not being able to report their orientation. We expected that, if central and peripheral regions segmented, then the CRIP effect would decrease. Therefore, we expect better discrimination of peripheral lines when the gap is bigger and when the two surfaces are less similar. The specific hypotheses and predictions are detailed in each experiment and summarized in Table 1.

**Table 1.** Description of the experiments. The type of distractors, the manipulations used, the tasks, and the expected results (prediction) are reported for each experiment. Experiment 5 was divided into two blocks with two different tasks, which are reported separately.

Exp	Distractors	Manipulation	Task	Prediction
1	×s, only grid, nothing	Target–distractor distance Distractor presence	Judge lines orientation ±45°	Worst performance when distractors are present and target–distractor distance is smaller
2	×s	Target eccentricity Number of distractors Gap position	Judge lines orientation ±45°	Worst performance for higher target eccentricity Worst performance for smaller target–distractor distance Worst performance for more peripheral gap position
3	Vertical lines	Target eccentricity Target–distractor distance Gap position	Judge lines orientation ±45°	Worst performance for higher target eccentricity Worst performance for smaller target–distractor distance or no differences in performance Worst performance for more peripheral gap position
4	Diagonal lines	Target eccentricity Target–distractor distance Gap position	Judge lines orientation ±45°	Same results as Experiment 2 or same results as Experiment 3 (depending on what makes vertical lines special)
5	×s	Target–distractor distance	Subjective report: What do you see in the periphery (lines, ×s, nothing)	More *×s* or *lines* than *nothing* responses
			Objective report: Judge lines orientation ±45°	Worst performance for smaller distance

**Table 2.** Post hoc comparisons for Experiment 5. The compared conditions are shown in the Contrast column. In the Contrast column, grid represents the condition without distractors, and grid + stim represents the condition with both grid and distractors. Big gap corresponds to a distractor eccentricity of 5.2° and small gap corresponds to 11.2°.

Contrast	Estimate	*SE*	*df*	*t* Ratio	*p*
Baseline vs. grid big gap	−0.0512	0.241	72	−0.213	0.8320
Baseline vs. grid small gap	0.0756	0.241	72	0.314	0.8320
Baseline vs. grid + stim big gap	1.0202	0.241	72	4.241	0.0001^*^
Baseline vs. grid + stim small gap	1.6601	0.241	72	6.901	<0.0001^*^
Grid big gap vs. grid small gap	0.1268	0.241	72	0.527	0.7997
Grid big gap vs. grid + stim big gap	1.0714	0.241	72	4.454	0.0001^*^
Grid small gap vs. grid + stim small gap	1.5845	0.241	72	6.587	<0.0001^*^
Grid + stim big gap vs. grid + stim small gap	0.6399	0.241	72	2.660	0.0154^*^

* Significant *p*-values.

**Table 3.** All of the different combinations of outer and inner border eccentricities and their resulting gap size. Outer border eccentricity is expressed in degrees from fixation to the farther border of the gap. Inner border eccentricity is from fixation to the closer border. The resulting gap sizes are expressed in degrees from one border to the other.

		Outer border eccentricity			
		7.2°	9.2°	11.2°	13.2°
Inner border eccentricity	5.2°	2°	4°	6°	8°
	7.2°	—	2°	—	6°
	9.2°	—	—	2°	4°
	11.2°	—	—	—	2°

### General methods

#### Participants

In Experiment 1, 19 participants were recruited. For Experiments 2 to 5, different samples of 12 participants were recruited. They were adults with normal or corrected-to-normal vision. At the start of each experiment, participants provided informed consent. The procedures were approved by the Ethics Committee of the University of Padua on psychological research (protocol number 4855). The study adhered to the tenets of the Declaration of Helsinki. Data for Experiments 2 to 5 were collected and analyzed between November 22, 2022, and November 15, 2023. Data for Experiment 1 were collected and analyzed between October 23, 2024, and December 1, 2024.

#### Stimuli

The experiment was programmed using Python and Psychopy (Peirce, 2009). The stimuli consisted of a grid with 2 degrees of visual angle (deg) wide square cells with diagonal lines in the center, as shown in Figure 1. In the central part, we used ×s for Experiments 1, 2, and 5; vertical lines in Experiment 3; and diagonal lines in Experiment 4. The diagonal lines, both the central and the peripheral ones, could be oriented at –45° or +45° with respect to the vertical axis and were 18 arcmin long. The ×s were created by adding both orientations. In this way, the ×s had a crossing point with double the luminance compared to their branches. This may not be noticeable in the figures, as we increased both contrast and luminance to make them more visible for the reader. The original stimuli can be found at https://osf.io/sy64w/. Both the grid and the lines were white (approximately 7.63 cd/m^2^) on a gray background (approximately 0.72 cd/m^2^). A central red cross of 30 × 30 arcmin was always present. Between the center and the periphery there was a gap. Except in Experiments 1 and 4, there were two types of grids. In 50% of the trials, the fixation cross was in the center of the central square (grid position off), and in the other 50% it was at the intersection of the four central squares (grid position on). The effect of grid position is discussed in the Supplementary Materials.

#### Procedure

**Figure 2.**
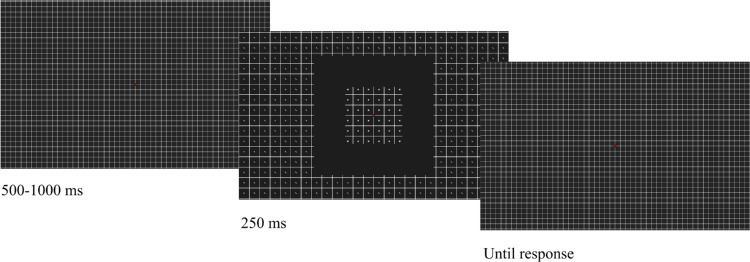
General procedure for all of the experiments described in this study. A fixation period of 500 to 1000 ms, randomly selected, was followed by a 250-ms stimuli presentation. During the fixation period and after stimuli presentation, we presented a grid in Experiment 5. In the other experiments, we presented only the fixation cross.

After a fixation time, the stimuli were displayed for 250 ms. The fixation time was randomly chosen in a range of 500 to 1000 ms, as illustrated in Figure 2. Animated versions of the stimuli of Experiments 1 to 4 can be found at the OSF link https://osf.io/sy64w/. The reader can get a general impression of how the stimuli looked in the periphery by fixating the red cross while judging the orientation of the targets. These stimuli are made to be displayed on large monitors, thus they may appear distorted when using small screens.

In Experiment 5, during fixation and after stimulus presentation, a square grid composed of the sum of the two possible grid positions was shown. In the other experiments, the screen was blank but for the fixation cross. In all of the experiments, participants were asked to report peripheral line orientations. There was no time limit and participants were encouraged to be accurate rather than fast. Participants responded by pressing keys on a keyboard. In Experiment 5, there was a block of trials before the discrimination task in which participants reported what they perceived in the periphery (×s, lines, or nothing).

The experiments were carried out in a dark room, and a chin rest was used to ensure a viewing distance from the screen of 57 cm. To be sure that participants did not make saccades away from fixation during stimuli presentation, a Gazepoint GP3 eye tracker (60 Hz; Gazepoint, Vancouver, BC, Canada) was used. All of the experiments were programmed to not show the stimuli if the participant was not fixating within 2° from the fixation cross. At the start of each trial, after a random interval between 500 and 1000 ms, there was a check if participants were fixating within the given 2°. If yes, the stimuli were shown; otherwise, after another 500 to 1000 ms an additional check was performed. Due to the random fixation time before stimuli presentation, participants could not build up any expectations as to when the stimulus would be shown. In this way, executing a saccade would require more time when the stimulus was displayed. Moreover, the stimuli duration was 250 ms. Although this is enough time to execute a saccade, saccade latency is generally increased when a stimulus is simultaneously presented at fixation, as in our experiments (Hermens & Walker, 2010). For these reasons, we did not register if any saccade was made during stimuli presentation.

## Experiment 1. Demonstrating the CRIP effect

We tested the existence of the CRIP effect by comparing participants’ performance in an orientation judgment task on peripheral informative lines (targets) with and without central non-informative elements (distractors). When the distractors were absent, the squared grid could be either present or absent. We also manipulated the distance between distractors and targets. We expected that the presence of distractors would impair performance in peripheral orientation discrimination. Specifically, we hypothesized the same level of performance in both distractor-absent situations (grid present and grid absent). Thus, only the presence of distractors would affect discrimination. We also expected that performance would further drop at smaller target–distractor distance.

### Methods

#### Participants

We recruited 30 participants (mean age = 20.97 years; *SD* = 1.83; 26 females, three males, and one non-binary). The overall accuracy was high, likely due to the presence of a baseline condition which, according to our hypotheses, would increase performance. To avoid ceiling effects, we excluded participants with an accuracy higher than 90% in the more difficult condition (distractors present and small target–distractor distance). The remaining sample was composed of 19 participants (mean age = 21.58 years; *SD* = 1.68; 16 females, two males, and one non-binary).

#### Apparatus

Stimuli were presented on a 31.5-inch LG UN500P monitor (LG Electronics, Seoul, South Korea) with a refresh rate of 60 Hz, resolution of 3840 × 2160 pixels, and size of 70 × 39.5 cm.

#### Design and procedure

**Figure 3.**
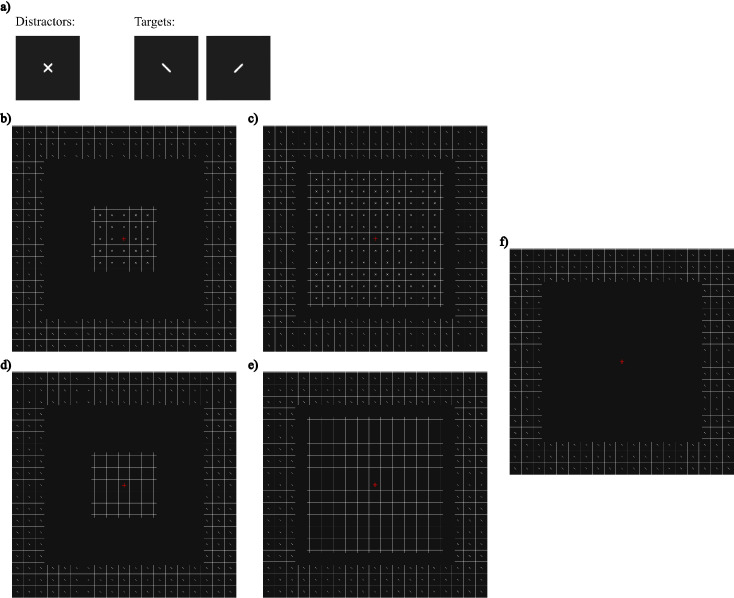
Stimuli and conditions for Experiment 1. (**a**) The targets and distractors used. The distractors are the elements in the center, and the targets are the elements in the periphery. Targets could be tilted ±45°. Distractors were composed by superimposing the two target orientations. (**b**, **c**) The conditions in which the distractors were present. In these conditions, two gap sizes were chosen: a large gap in which target–distractor distance was bigger (**b**), and a small gap in which the distance was smaller (**c**). In both conditions, the eccentricity of the targets was fixed, so the only change was the eccentricity of distractors. (**d**, **e**) The same gap sizes were used for the conditions in which only the grid without distractors was present. (**f**) The baseline condition, without grid nor distractors. In this figure, the grid has been cropped and its contrast increased for better resolution. In the experiment, the grid extended farther into the peripheral visual field and the contrast was lower.

In Experiment 1, we presented three types of stimuli: a baseline, with no grid and no distractors in the center; a condition in which only the grid was present without distractors (grid); and a condition in which both the grid and the distractors were present (grid + stim). For the last two types of stimuli, the gap dividing the center from the periphery had two sizes (small and large). Figure 3 shows the stimuli used in this experiment.

Target eccentricity was fixed at 13.2°. Distractor eccentricities were 5.2° (big gap: 6° of size) and 11.2° (small gap: 2° of size). Eccentricity was measured from the fixation cross. We only used the off grid position, in which the fixation cross was placed inside one of the squares of the grid.

The experimental design was 2 (line orientations) × 5 (baseline, grid with large and small gaps, grid + stim with large and small gaps). Each condition was repeated 14 times for a total of 140 trials. Before the experimental block, participants completed a practice block (20 trials, each condition repeated twice) with acoustic feedback for right and wrong answers. In the experimental block no feedback was provided.

### Analysis

For each condition we extracted the *d’*. We used the following formula:

$$\begin{array}{c} d^{'}=\;\frac{Z\left(\mathrm{leftCorrect}\right)-Z\left(\mathrm{leftIncorrect}\right)\;}{\sqrt{2}} \end{array}$$

In the formula, *leftCorrect* represents the rate of correct responses for the –45° targets (when the targets orientation was –45° and the participant correctly reported –45°) and *leftIncorrect* represents the rate of incorrect responses for the +45° targets (when targets orientation was +45° and the participant incorrectly reported –45°). Higher *d’* corresponded to higher performance. A *d’* equal to zero corresponds to an accuracy of 50% (chance level).

We conducted a linear mixed-model ANOVA with *d’* as the dependent variable, condition as the independent variable, and participant as the random factor. We then conducted post hoc pairwise comparisons with correction for multiple comparisons (false discovery rate). Considering the high number of participants excluded due to being at the ceiling in the most difficult condition (small gap with distractors present), we also analyzed the sample without exclusions. These results are reported in the Supplementary Materials.

### Results

**Figure 4.**
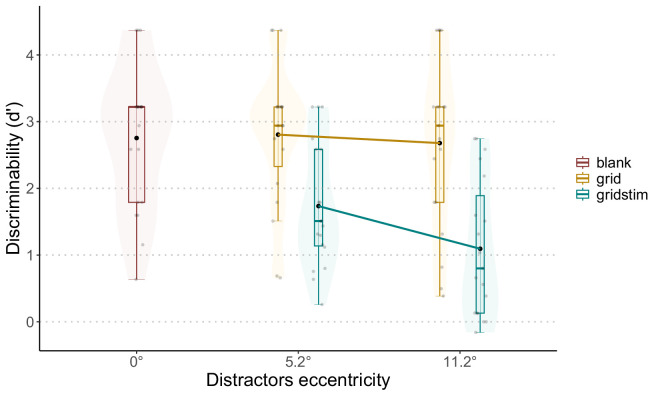
Performance for every condition in Experiment 1. *d’* is plotted as a function of the distractor eccentricity. The 0° eccentricity (blank in the legend) corresponds to the baseline condition, with nothing in the center. The label “grid” represents the condition in which the grid was present without distractors, and “gridstim” stands for the presence of both grid and distractors. Black dots represent the averages for each condition.

Figure 4 shows *d’* as a function of distractor eccentricity and elements in the center. Another version of these plots, with different colors for each participant, can be found in the Supplementary Materials. The ANOVA revealed an effect of condition, *F*(4, 72) = 20.238, *p* < 0.01. Post hoc pairwise comparisons are reported in Table 2. The baseline was significantly different from both conditions in which the distractors were present. No significant differences were found between the baseline and the two grid conditions. The difference between the two gap sizes in the grid conditions was not significant. Considering the gap sizes, both differences between the grid conditions and the grid + stim conditions were significant (small gap grid vs. grid + stim, large gap grid vs. grid + stim). The difference between the two gap sizes for the grid + stim conditions was also significant.

### Discussion

In Experiment 1, we demonstrated the existence of the CRIP effect, as elements in the center (distractors) impaired discrimination of elements in the periphery (targets), and the impairment was stronger at smaller target–distractor distances. We demonstrated that the CRIP effect does not occur when only the grid is shown in the center. This suggests that the effect is related to the similarity between targets and distractors. In Experiment 1, the ×s in the center were designed by superimposing both targets orientations. In Experiments 3 to 4, we compared the effect of different types of distractors on peripheral discrimination. In Experiment 2, we controlled for target eccentricity.

## Experiment 2. Comparing the effect of distractor and target eccentricity

**Figure 5.**
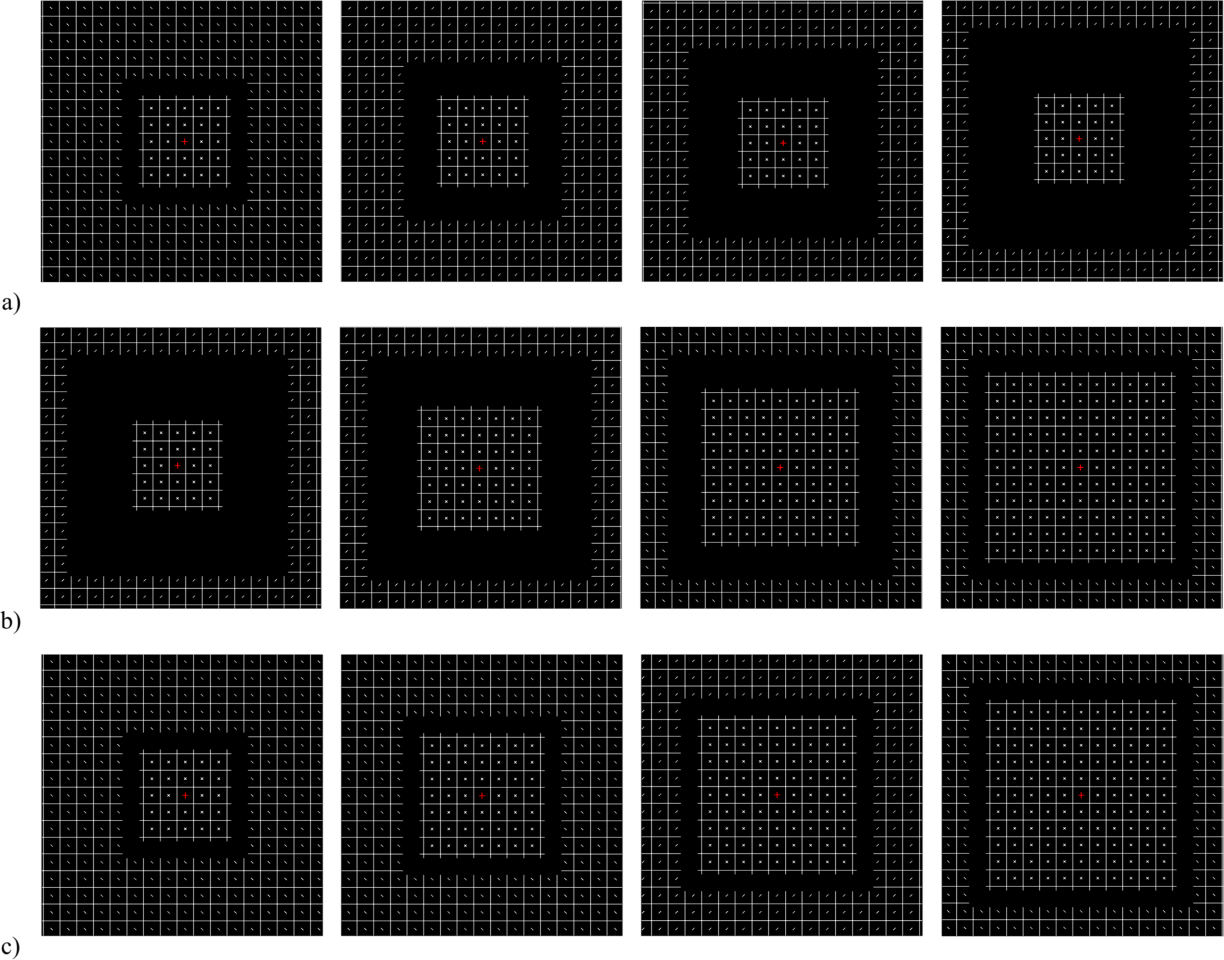
The three manipulations used in Experiment 2. (**a**) Target eccentricity manipulation: The number of distractors was fixed but the gap size changed. This manipulation allowed controlling for the variation in starting points of the peripheral targets. (**b**) Distractor manipulation: The eccentricity of the targets was fixed, but the size of the gap changes allowed controlling for the varying target–distractor distances and number of distractors. (**c**) Gap position manipulation: The size of the gap (target–distractor distance) was fixed at 2°, but both the number of distractors and target eccentricity changed. In the figure, the grid has been cropped in the periphery and its contrast has been increased. In the experiment, the grid extended farther into the peripheral visual field and the contrast was lower.

In Experiment 2, we kept the same targets and distractors as in Experiment 1, but we added two manipulations: target eccentricity and gap position (Figure 5). In the target eccentricity manipulation, the eccentricity of distractors was fixed, and we varied the target–distractor distances by varying the size of the gap, which led to a more peripheral presentation of the targets. The manipulation was aimed at comparing the magnitude of both effects: distractor number and target eccentricity. In the gap position manipulation, gap size (and thus target–distractor distance) was fixed, but the outer and inner borders changed. This manipulation allowed us to investigate the effect of distractors and target position when the distances were fixed.

We expected to replicate results from Experiment 1 for the distractor manipulations. For the target eccentricity manipulations, we expected worse performance when the targets were more peripheral. For the gap position manipulation, we expected a decrease in performance when the gap was more peripheral. In this condition, both the number of distractors and target eccentricity increased, but the target–distractor distance was fixed. We expected that the number of distractors would have an effect on performance.

### Methods

#### Participants

For this experiment, 12 participants were recruited (mean age = 21.25 years; *SD* = 2.31; 10 females, one male, and one non-binary). These participants did not participate in Experiment 1.

#### Apparatus

The same apparatus as Experiment 1 was used.

#### Design and procedure

We manipulated the gap size according to two variables: inner and outer border of the gap. They can be described as the eccentricity of the closer and farther borders of the gap. Inner and outer borders determined nine different combinations, which are illustrated in Table 3. The design was a 2 (lines orientation ±45°) × 9 (combinations of outer and inner borders) × 2 (on vs. off grid position) factors for a total of 36 conditions. Participants reported the orientation of peripheral lines (±45°) by pressing a key. Each stimulus was repeated 14 times for a total of 504 trials. Participants completed a practice block (72 trials) with acoustic feedback for right and wrong answers. In the experimental block, no feedback was provided.

### Analysis

We extracted *d’* values using the same formula as in Experiment 1. We conducted a mixed-model type III ANOVA with Satterthwaite's method for each manipulation, so we had three models. The random factor was the participant, and the fixed factor was the manipulation (target eccentricity, distractors, or gap position). We then extracted the standardized coefficients of the fixed factor for each model to see which manipulation was more effective.

### Results

**Figure 6.**
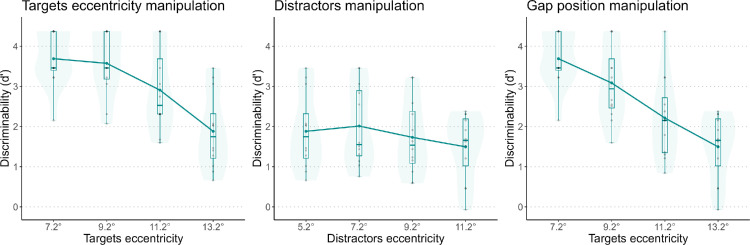
*d’* for the three manipulations. Each plot represents one manipulation. Each dot represents one participant, and the black dots represent the averages.

Figure 6 shows the effect of each manipulation on performance. Another version of these plots, with different colors for each participant, can be found in the Supplementary Materials. For each model, we found a significant effect of the manipulation: target eccentricity, *F*(1, 35) = 42.208, *p* < 0.01; number of distractors, *F*(1, 35) = 5.094, *p* = 0.30; and gap position, *F*(1, 35) = 73.628, *p* < 0.01. The standardized coefficients were –6.497 for target eccentricity, –2.257 for distractor eccentricity, and –8.580 for gap position.

### Discussion

We replicated and extended findings from Experiment 1. Together with results from Experiment 1, these results suggest a general effect of the presence of distractors in the center. More specifically, increasing the number of distractors and decreasing their distance from the target impaired performance in orientation discrimination tasks. Increasing target eccentricity also impaired performance. This was an expected result, given that the ability to discriminate objects decreases with eccentricity. Nonetheless, it is interesting to note that the effect of target–distractor distance is modulated by target eccentricity. If performance were only dependent on target eccentricity, then the effect size for gap position would be exactly the same as the effect size for target eccentricity (see Figure 5). The coefficient comparison suggests that the number of distractors affects performance and contributes to the effect, which is therefore not entirely due to target–distractor distance.

## Experiment 3. Manipulating distractor orientation: Using vertical lines

In previous experiments, distractors were ×s. In Experiment 3, we used single vertical lines. We expected that, if the task-related information (or target–distractor similarity) is an important factor in the CRIP effect, then changing target–distractor distance or increasing the number of distractors would not affect performance, whereas target eccentricity would affect it. Hence, we expected no effect of distractor manipulation on performance for vertical lines.

### Methods

#### Participants

In this experiment, 12 participants who did not participate in Experiments 1 or 2 were recruited. Three participants performed at ceiling in five out of the nine combinations and were excluded for this reason. We recruited three additional participants to keep the sample equal to Experiment 2. Hence, the final sample was composed of 12 participants (mean age = 24.00 years; *SD* = 10.81; 10 females and two males).

#### Apparatus

The same apparatus as in Experiments 1 and 2 was used.

#### Design and procedure

The same design and procedure of Experiment 2 were used. The only difference was in the nature of the central distractors, as we used single vertical lines instead of ×s.

### Analysis

We conducted the same analysis as in Experiment 2.

### Results

**Figure 7.**
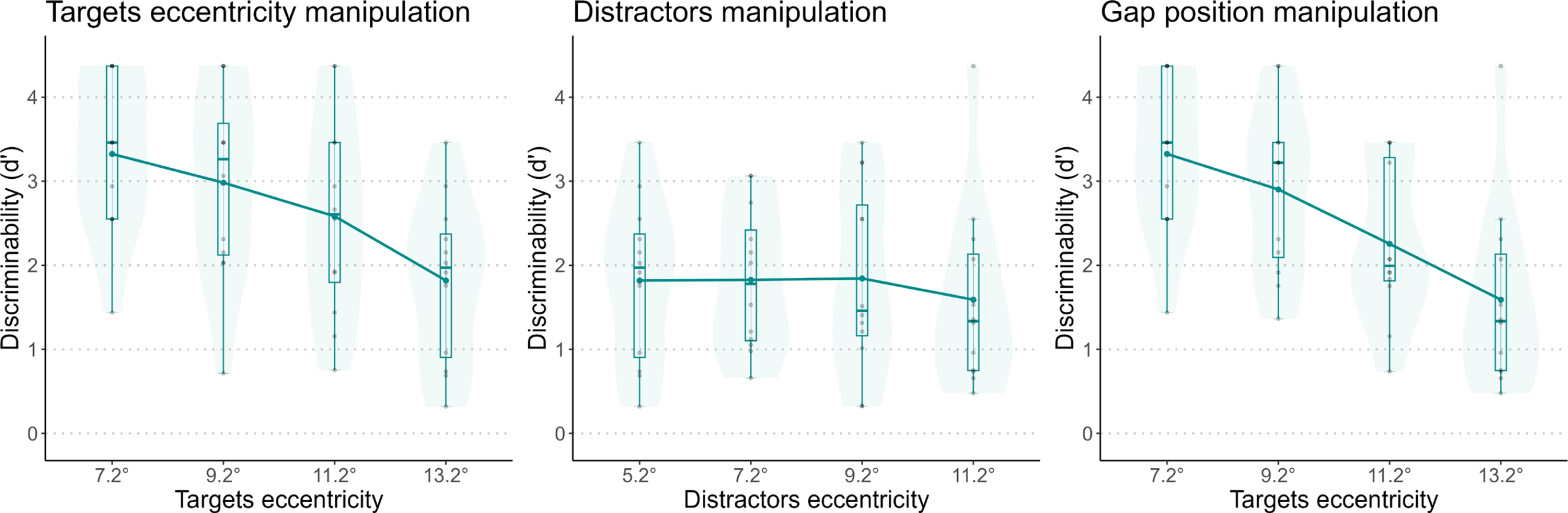
*d’* for the three manipulations. Each plot represents one manipulation. In the gap position manipulation, both target and distractor eccentricities varied.

Figure 7 shows the effect of each manipulation on performance. Another version of these plots, with different colors for each participant, can be found in the Supplementary Materials. We found a significant effect of target eccentricity, *F*(1, 35) = 33.705, *p* < 0.01. In the distractor manipulation, there was no significant effect of the number of distractors, *F*(1, 35) = 1.028, *p* = 0.318. In the gap position manipulation, we found a significant effect, *F*(1, 35) = 43.038, *p* < 0.01. The standardized coefficients were –5.804 for target eccentricity, –1.013 for distractor eccentricity, and –6.560 for gap position.

### Discussion

We did not find a significant effect of manipulating the number of the central distractors if the distractors were single vertical lines. Nevertheless, the effect of the gap position manipulation was stronger than that of target eccentricity alone, as observed in the standardized coefficients. This suggests that central vertical lines may enhance the effect of target eccentricity on peripheral discrimination. This effect was not strong enough to impact performance when the only manipulation was the number of distractors. This could be due to two reasons. The first one is that the vertical lines did not provide any task-relevant information on orientation. In this scenario, the ×s could be considered as the sum of the two orientations that peripheral lines could have, thus they were more disruptive. The second possibility is that the ×s had twice the information and luminance compared to the single lines. For this reason, they were stronger distractors (Rashal & Yeshurun, 2014). To disentangle the two possibilities, we conducted Experiment 4.

## Experiment 4. Manipulating distractor orientation: Using diagonal lines

In Experiment 4, distractors were single lines with the same or opposite tilt compared to the lines in the periphery. If the CRIP effect depends on task-relevant distractor orientations, we would expect results comparable to those for Experiment 2. That is, we expected an effect of number of distractors and target–distractor distance when target eccentricity was fixed. If the CRIP effect is mediated by the luminance of the distractors, then we could expect results similar to those of Experiment 3—that is, no effect of the number of distractors or target–distractor distance. In addition, this experiment tested the role of congruency in orientation between distractors and targets.

### Methods

#### Participants

In this experiment, 12 participants who did not participate in the previous three experiments were recruited. This experiment was more difficult than Experiments 2 and 3. To keep the overall *d’* as close as possible to the previous experiments and to avoid floor effects, we added an exclusion criterion of *d’* < 0.6. Four observers did not meet the criteria and were replaced by additional four participants to reach a sample size of 12 (mean age = 22.67 years; *SD* = 4.71; nine females, one male, and two non-binary).

#### Apparatus

The same apparatus as Experiments 1–3 was used.

#### Design and procedure

The same procedure and a similar design as Experiments 2 and 3 were used. We used single oriented (±45°) lines instead of ×s or single vertical lines. This resulted in an additional factor in the factorial design. To keep the same number of trials as previous experiments, we removed the grid position condition. We used the *off* position—that is, the one in which the fixation cross was in the center of the central square. Hence, the design was a 2 (line orientations) × 9 (conditions) × 2 (congruent vs. incongruent distractors) factors for a total of 36 conditions. Each trial was repeated 14 times for a total of 504 trials. The practice block (72 trials) had feedback for correct and incorrect responses. In the experimental block, no feedback was provided.

### Analysis

We analyzed the bias (criterion, *c*) toward responding according to the elements in the center. The *c* was calculated with the function dprime() of the psycho package (Makowski, 2018), which uses the following formula:

$$\begin{array}{c} c=\frac{-z\left(\mathrm{corrI}\right)+z\left(\mathrm{errC}\right)}{2} \end{array}$$

where *corrI* is the rate of correct responses for the incongruent condition, and *errC* is the rate of incorrect responses for the congruent condition. A negative *c* suggests a preference toward responding –45° when central lines are +45° oriented and +45° when central lines are –45° oriented (incongruency bias). A positive *c* would suggest the opposite (congruency bias). Zero means no bias. Having found an incongruent bias, we split the data into two groups: For each participant, we separated the congruent and incongruent orientation trials, thus creating a congruent group with 12 participants and an incongruent group with the same participants. We then performed the same analysis as in Experiments 2 and 3 for each group. It should be noted that the *d’* extracted when splitting the data into a congruent and incongruent condition cannot be disentangled from bias. For this reason, this *d’* cannot be considered a measure of discriminability per se, for it represents only the rate of congruent and incongruent responses.

Additionally, we calculated *c* as the tendency to report a specific orientation in two other ways. First, we substituted in the formula *corrI* with the rate of correct responses for the –45° orientation and *errC* with the rate of incorrect responses for the +45° orientation, independent of the congruency. This analysis is reported in the Supplementary Materials. Second, we split those data into two groups according to the orientation of the distractors (a –45°-oriented distractor and a +45°-oriented distractor). We then extracted the corresponding *d’*. Again, this *d’* cannot be disentangled from bias and thus cannot be considered a measure of discriminability. In both groups, this *d’* represents only the rate of –45° responses.

### Results

**Figure 8.**
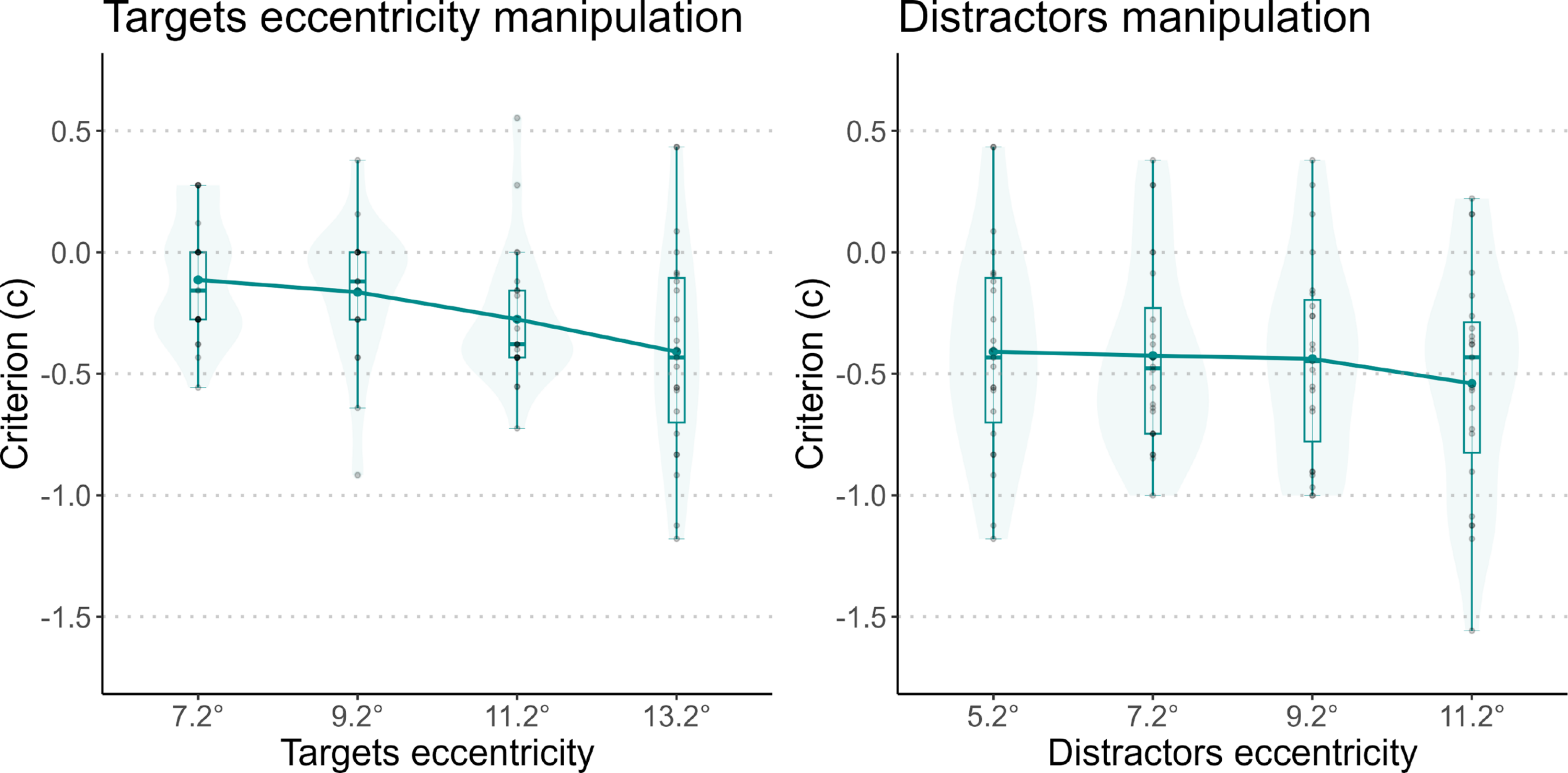
Criterion variation. On the left panel, the criterion is plotted as a function of target eccentricity. On the right panel, it is plotted as a function of distractor eccentricity.

Figure 8 shows the *c* values for the target and distractor manipulations. Another version of these plots, with different colors for each participant, can be found in the Supplementary Materials. The *c* values were negative for both manipulations, indicating an overall incongruency bias. When calculating *c* based on the rate of correct –45° and incorrect +45° responses, it was close to zero. When calculating it splitting the data based on distractor orientation, the incongruency bias was confirmed. These results are reported in the Supplementary Materials.

**Figure 9.**
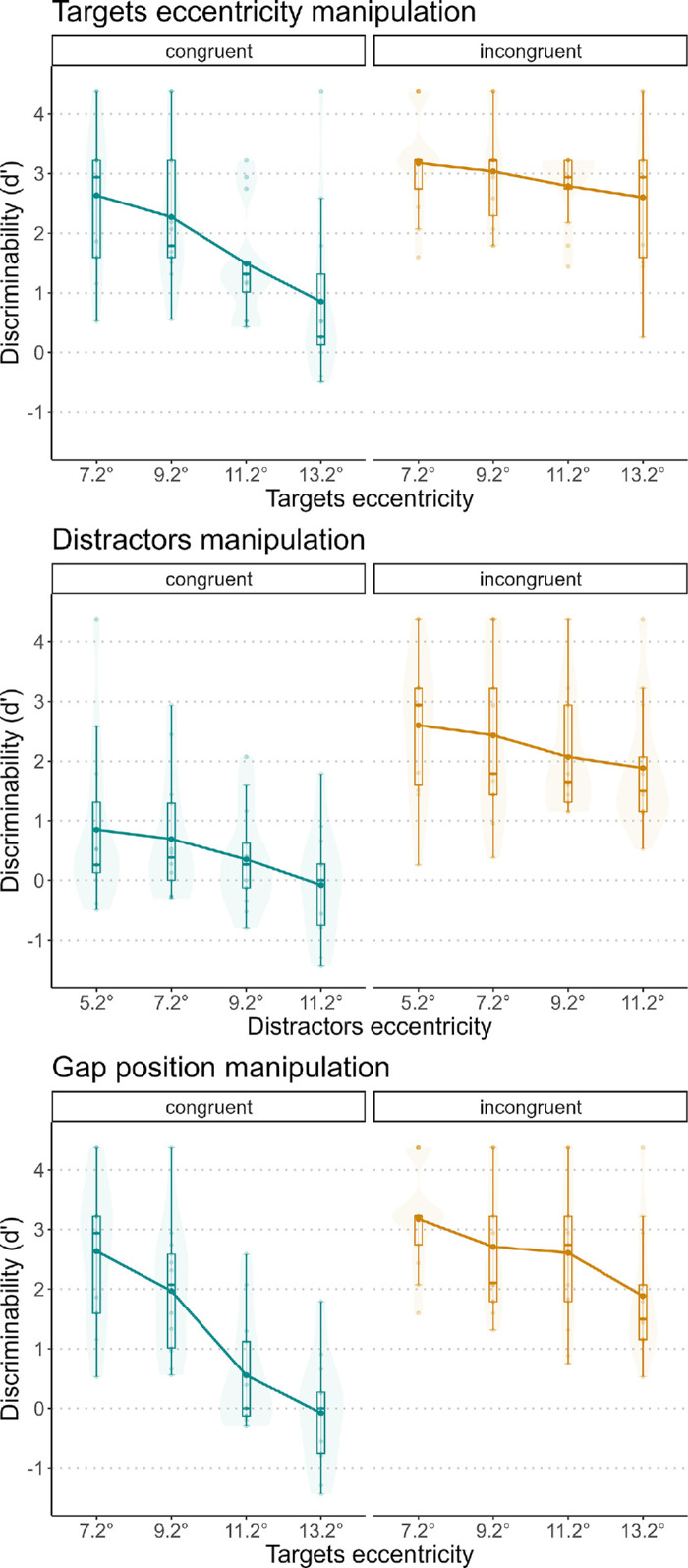
*d’* for the three manipulations, divided for congruent and incongruent conditions. Each plot represents one manipulation. In the gap position manipulation, the eccentricities of both targets and distractors changed.

Figure 9 shows the *d*’ values for each manipulation separately for the congruent and incongruent groups. Another version of these plots, with different colors for each participant, can be found in the Supplementary Materials. The average *d’* for the congruent condition was 0.764, and it was 1.883 for the incongruent condition. For the congruent condition, the mixed-model ANOVAs on the three manipulations confirmed significant effects: target eccentricity, *F*(1, 35) = 27.893, *p* < 0.01; distractors, *F*(1, 35) = 17.483, *p* < 0.01; and gap position, *F*(1, 35) = 71.774, *p* < 0.01. We also found significant effects for all the manipulations in the incongruent condition: target eccentricity, *F*(1, 35) = 4.670, *p* = 0.37; distractors, *F*(1, 35) = 7.168, *p* = 0.11; and gap position, *F*(1, 35) = 16.394, *p* < 0.01.

Similar results were found when the data were divided for the orientation of the central lines. For the –45° group, all three models revealed a significant effect of the variable taken into account: target eccentricity manipulation, *F*(1, 35) = 22.887, *p* < 0.01; distractor manipulation, *F*(1, 35) = 6.644, *p* = 0.14; and gap position, *F*(1, 35) = 51.264, *p* < 0.01. The same results occurred for the +45° group: target eccentricity manipulation, *F*(1, 35) = 11.291, *p* = 0.02; distractor manipulation, *F*(1, 35) = 15.323, *p* < 0.01; and gap position, *F*(1, 35) = 67.631, *p* < 0.01.

### Discussion

Our results confirm that the CRIP effect depends on the orientation of distractors, as we found results similar to those of Experiment 2. Specifically, we found that the central oriented lines acted as distractors in an orientation discrimination task on peripheral oriented lines in the same way as central ×s. We can conclude that for the CRIP effect to occur distractors have to be similar to the targets, at least in orientation.

Overall, *d’* was higher in the incongruent condition, meaning that the CRIP effect is overall stronger in the congruent condition. However, a distinction should be made between the effect of the presence of distractors and an effect of target–distractor distance. As demonstrated in Experiment 1, the presence of distractors is enough to impair discrimination. This holds true for both ×s and diagonal lines. In Experiment 4, participants had a lower *d’* in the congruent condition even at lower eccentricities, whereas in the incongruent condition *d’* decreased only at larger eccentricities. This can be explained by a bias toward responding the opposite of the central orientation (incongruency bias). An explanation for this bias could be that participants were not be able to extract the real orientation of the peripheral lines, but they perceived a difference between central and peripheral elements. Because the only response options in Experiment 4 were ±45° (that is, the same and the opposite orientation as the central elements), participants responded with the opposing orientation when they just perceived a different pattern between the center and periphery. Another reason behind the difference in performance between the congruent and incongruent conditions may be related to segmentation. Opposing orientations help segmentation, thus rendering peripheral lines more visible. As a result, the difference in performance is not related to a bias, because participants can actually perceive the peripheral lines better when there is more segmentation. It was impossible to distinguish between the two explanations due to the experimental design. The incongruency bias should be addressed in future studies, using a similar task as in Experiment 4 but including more response options—for example, giving the participant the possibility of responding “I perceived diagonal lines but I do not know their orientation.”

Whatever the reason behind this bias is, results from Experiment 4 are counterintuitive, as they showed that, when targets and distractors had the same orientation, performance was worse. They also go against what one might expect based on the principle of extrapolation (Stewart, Valsecchi, & Schütz, 2020). We conclude that, in the CRIP effect, there is no tendency to extrapolate what is in the center to the periphery.

## Experiment 5. Subjective report

In Experiment 5, we controlled for the transient onset of the stimuli by adding a grid between stimuli presentations (see Figure 2). We also collected subjective and objective reports to probe a possible dissociation between phenomenal experience and performance in extrapolation. We expected that smaller target–distractor distance (higher number of distractors) would impair the discriminability of the targets (CRIP effect) even when the onset of the stimuli was not transient. When participants reported what they perceived in the periphery (see subjective report in Table 1), two scenarios were possible. In the first, participants could see the peripheral elements and identify their shape; hence, we would expect mainly *line* responses. In the second scenario, participants saw peripheral elements but could not identify their shape; in this case, we would expect extrapolation and therefore more *×* responses. Independently of which characteristic can be extracted, we expected few *nothing* responses.

### Methods

#### Participants

Twelve participants who did not participate in previous experiments were recruited (mean age = 22.42 years; *SD* = 1.88; nine females and three males).

#### Apparatus

Stimuli were presented on a 24.1-inch Eizo CS2420 LCD monitor (Eizo Global, Hakusan, Japan) with a refresh rate of 60 Hz, resolution of 1920 × 1200 pixels, size of 51.8 × 32.4 cm, color temperature of 6500K, and luminance resolution of 10 bits per color channel.

#### Design and procedure

We used the same targets as in previous experiments and ×s as distractors. We used only the distractor manipulation. The experimental design was a 2 (line tilts) × 2 (9.2° vs. 5.2° distractor eccentricity) × 2 (on vs. off grid position) between-subjects factors. A distractor eccentricity of 9.2° corresponded to a gap size of 2°. A distractor eccentricity of 5.2° corresponded to a gap size of 6°. The eccentricity of the targets was 11.2°. Eccentricity was measured from the fixation cross.

Each participant completed two blocks, with the same stimuli but different tasks. In the first block, they reported what they saw in the periphery: ×s, lines, or nothing. Participants were instructed to report *nothing* when they saw the grid in the periphery but without any elements inside the squares. Given that we wanted participants to give a more subjective report on the shape of the peripheral elements, we gave them three options. Participants reporting *lines* meant that they could correctly extract the shape of the stimuli. On the other hand, a higher number of *×* responses would mean that participants extrapolated the central elements to the periphery, assuming uniformity of the texture. If participants reported seeing neither lines nor ×s but just the grid, it would mean that the central elements masked the perception of the overall shape of the peripheral elements. It could also mean that the eccentricity of the peripheral elements was too large or their lengths were too short. In this sense, the *nothing* option allowed us to test if participants were able to perceive elements other than the grid in the periphery. If they could not perceive the elements, giving them the possibility to choose only between lines and ×s without adding this sort of control option could have led to misleading results, in which participants were responding at chance. In the second block, they reported the orientation of the peripheral lines (±45°). Each stimulus was repeated 40 times, for a total of 320 trials per block. Before each experimental block, there was a practice block to familiarize participants with the task. The practice block preceding block 1 had 32 trials (each condition repeated once). The practice block preceding block 2 had 64 trials (each condition repeated twice) and had acoustic feedback for right and wrong answers. For the experimental blocks and for the practice block preceding block 1, no feedback was provided.

### Analysis

For block 1, we conducted multinomial logistic regressions using the multinom() function of the R package nnet (Ripley & Venables, 2023). Response (×s, lines, or nothing) was the categorical dependent variable. We compared a null model without predictors with the model that included distractor manipulation, treated as a factor with two levels. The best model selection was made with the Bayesian information criterion for a more conservative approach. It imposes strict penalties for added model complexity, thereby promoting the selection of parsimonious models. After selecting the best model, we extracted the log odds of each response category. The response *lines* was the reference. Negative coefficients represent a decrease in the response (×s or nothing), and positive coefficients represent an increase. For block 2, we extracted the *d’* as in Experiments 1 to 3, then we conducted a linear mixed-model ANOVA with *d’* as the dependent variable, distractor eccentricity as a categorical variable with two levels, and participant as the random factor.

### Results

**Figure 10.**
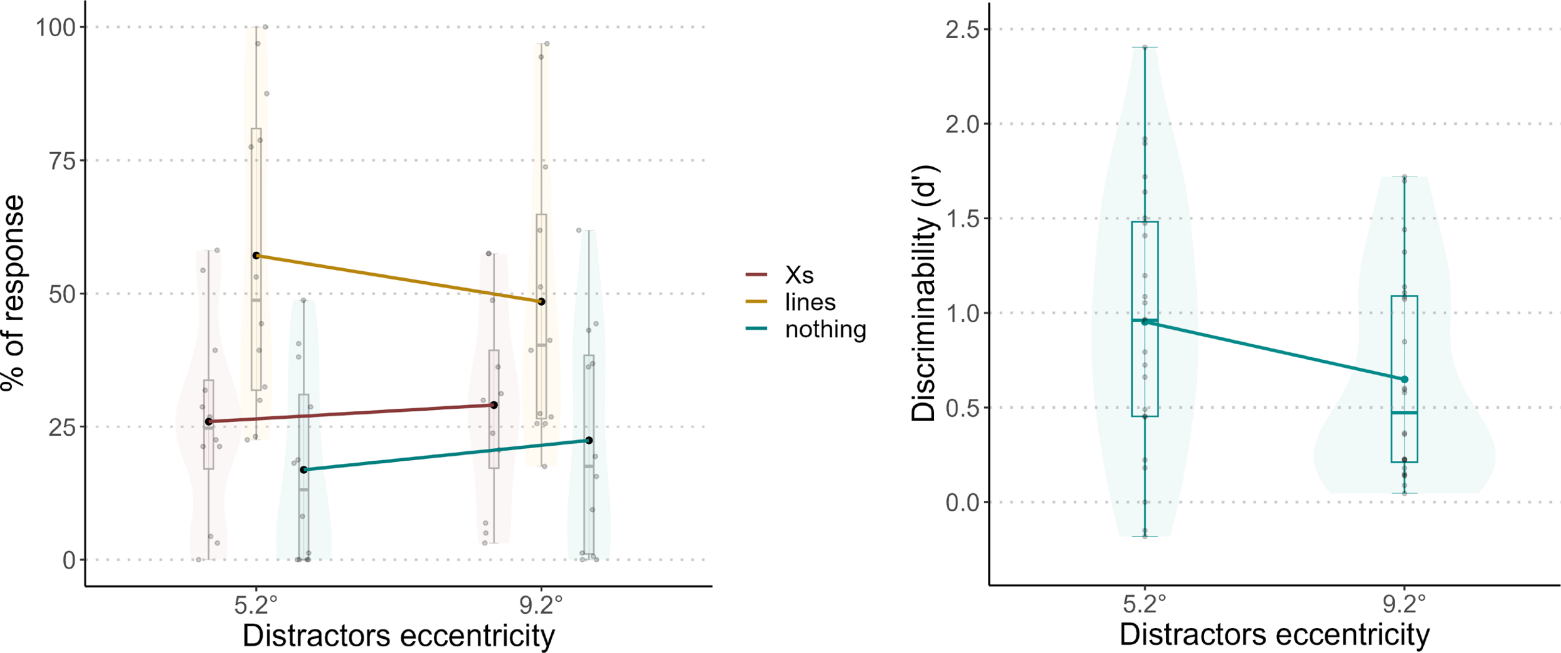
Results from block 1 (left panel) and block 2 (right panel). The left panel shows the percentage of responses for each category (*×s*, *lines*, or *nothing*). The responses are divided for the eccentricities of the two distractors. The right panel shows the *d’* value for each distractor eccentricity. For both plots, each dot represents a participant, and the black triangles represent the averages.

Figure 10 shows the results from block 1 (left panel) and block 2 (right panel). Another version of these plots, with different colors for each participant, can be found in the Supplementary Materials. For the 5.2° eccentricity, the percentage of *lines* responses was 57.1%, the percentage of ×s responses was 26.0%, and the percentage of *nothing* responses was 16.9%*.* For the 9.2° eccentricity, the average percentage of *lines* responses was 48.5%, the percentage for *×s* was 29.1%, and the percentage for *nothing* was 22.4%. For block 1, the model with distractor eccentricity as a predictor was better than the null model (Table 4).

**Table 4.** Model selection for block 1. For each model, we report the Bayesian information criterion (BIC). The smaller the BIC, the better the fit. The null model represents the model without any factor. Distractor eccentricity represents the model with distractor eccentricity as a factor. *K* is the number of parameters in the mixed model including fixed and random effects. Delta BIC refers to the difference in the BIC scores between the current model and the best model.

Model	*K*	BICc	Delta BIC
Distractor eccentricity	4	7772.99	0.00
Null	2	7788.44	15.45

We then analyzed the coefficients of the selected model. When the eccentricity increased from 5.2° to 9.2°, the coefficient for the *×s* response increased by 0.278, whereas the coefficient for the *nothing* response increased by 0.447. Having both positive coefficients suggests that participants chose *lines* more times than the other two responses when distractor eccentricity was 5.2°. In block 2, for the 5.2° eccentricity the average *d’* was 0.94, and for the 9.2° eccentricity the average *d’* was 0.64. The ANOVA on the linear model revealed a significant effect of distractor eccentricity, *F*(1, 35) = 13.043, *p* < 0.01.

### Discussion

In Experiment 5 we replicated the CRIP effect, for the performance was higher when target–distractor distance was larger. Results from block 1 suggest the ability to perceive the shape of peripheral targets. The percentage of reported *lines* decreased with the number of distractors, suggesting that the participants’ perception was impaired when distractors were added or when they were closer to the targets. Note that this response in the first task could also be explained as a bias; the participants may simply have said *lines* when unsure. Block 2 measured sensitivity. The explanation based on distractors interference is corroborated by this discrimination task: The lower the number of distractors (or the farther away from the targets), the higher the sensitivity (*d'*). Together, our findings confirm the existence of the CRIP effect.

As reported in the General methods section, in this experiment a square grid was shown during fixation and after stimulus presentation. This may have resulted in an afterimage lasting even when the stimuli were presented. We verified that this was not the case, however, by trying the experiments ourselves and administering them to other volunteers within the lab before starting the data collection. Moreover, we asked participants to make some notes on the experiments after completing them, but none of the participants reported an afterimage. On the other hand, it is still possible that the presence of the grid affected the performance, as easily seen when comparing the results from Experiments 2 and 5. However, the CRIP effect occurred regardless of the fixation grid. Participants’ performance was lower in Experiment 5 compared with Experiment 2 (without the grid between the stimuli). We can conclude that the presence of the grid during fixation impaired general performance but did not contribute to the CRIP effect.

## General discussion

In this study, we investigated a new phenomenon, which we refer to as the CRIP effect. We define the CRIP effect as the inability to extract information from peripheral elements as a result of interference from elements present in the central region. We explored the phenomenon in five experiments, using subjective and objective measures.

Our study was motivated by the following considerations. When there is a non-uniform stimulus extending from the fovea to periphery, at least three possible situations can occur. First, central and peripheral regions are perceived and analyzed independently; that is, there is no effect of the central region on the peripheral one. In the second scenario, the center and periphery are integrated by, for example, extrapolation, collinear peripheral facilitation (Maniglia, Pavan, Aedo-Jury, & Trotter, 2015), or luminance spreading (Watanabe & Sato, 1989). Note that effects of facilitation (affecting performance) and effects of extrapolation (affecting appearance) may dissociate. Finally, it is also possible that there is interference. As in crowding, this interference may be modulated by similarity: If the stimuli in the two regions are very different, there is less interference.

We demonstrated the existence of the CRIP effect in Experiment 1, ruling out the first scenario. The presence of elements in the center impaired peripheral discrimination, but only when the central and peripheral elements were similar. The closer the targets and distractors were the worse the performance. Note that in this set of experiments small gaps had reduced distances between distractors and targets, but there was also a larger total number of distractors. Separating these two factors will require further experiments.

In Experiment 2, we replicated the results of the first experiments and extended them by testing the effect of target eccentricity (not related to the CRIP effect) and the cumulative effect of target eccentricity and number of distractors. In Experiments 3 and 4, we manipulated the characteristics of the distractors. We used single lines to avoid the problem that an ×, as used in Experiments 1 and 2, has more pixels and more luminance than a single line. The CRIP effect was confirmed when the lines had one of the possible orientations that targets could have (that is, when they were diagonal and not vertical), and it was stronger when distractors and targets had the same orientation. This bias could be due to the fact that opposing orientations help with grouping the peripheral elements, reducing uncertainty. It can also be a case of iso-orientation suppression. Regardless of the reason behind the bias, it suggests that we do not rely on central vision to disambiguate elements in the periphery.

Experiment 4 particularly showed that the CRIP effect is different from other mechanisms occurring in patterns, such as extrapolation over time (as in the healing grid) and the uniformity illusion, where peripheral elements are perceived similarly to central ones even though they are different. These results argue against simple versions of predictive processing and suggest that the CRIP effect is similar to the honeycomb illusion (Bertamini, Oletto, & Contemori, 2024) and the extinction illusion, where homogeneous displays are perceived as different.

Experiment 5 showed that participants were able to detect elements in the periphery (lines) and perceive their shape, despite the impairment in discriminating their orientation at smaller target–distractor distances. Our study recorded two types of responses: phenomenal experience and objective discrimination. On the phenomenal level, participants choose *lines* more times than *×s* or *nothing*. They did not rely on central vision to guess about the periphery because the *×s* responses were few (no extrapolation). By comparing subjective and objective reports, we demonstrated that the phenomenal and objective levels are informative about different aspects of the phenomena.

In all of the experiments, we observed that central elements influence the extraction of features of the peripheral elements; that is, they impair orientation discrimination. Taken together, these results demonstrate that, in some cases, central vision may be disrupting peripheral discrimination, thus confirming the third scenario. Moreover, the CRIP effect supports the importance of peripheral vision in scene perception and its role as something more than just a lower resolution vision (Rosenholtz, 2023).

There are similarities between crowding and CRIP effect. In both CRIP and crowding, discrimination, but not detection, of the target is affected (Levi, 2011; Parkes, Lund, Angelucci, Solomon, & Morgan, 2001; Tripathy & Cavanagh, 2002), and similarity between targets and distractors modulates performance (Whitney & Levi, 2011). However, crowding is about peripheral–peripheral interactions, whereas in CRIP we are talking about center–peripheral interactions. For crowding, flankers must be rather close to the target, per Bouma's law (Bouma, 1970), whereas in the CRIP the minimum distance was 2°. Finally, although global properties of the entire stimulus layout are crucial in both crowding and the CRIP effect, when distractors form a regular pattern (e.g., because they are repeated), the crowding effect disappears (Herzog, Sayim, Chicherov, & Manassi, 2015). In the CRIP case, distractors always form a regular extended pattern.

Herrera-Esposito, Coen-Cagli, and Gomez-Sena (2021) demonstrated texture crowding. They presented textures in the periphery composed of a central and a surrounding area, either divided by a gap or continuous. When the gap was present, discrimination of the central texture was higher. The authors concluded that this represents a form of crowding, in which peripheral textures acted as flankers. Our results are in line with theirs; yet, there are important differences. First, they presented textures in the periphery. As a result, they found an effect specific to peripheral vision. In our experiments, patterns covered a large portion of the visual field, and the gap was larger. Second, they measured the effect of flankers on target elements, whereas our study focused on the effect of central vision on peripheral discrimination.

Future experiments should test the CRIP effect with different kinds of gaps. For example, a gap perceived as an occluder may increase the interaction between regions perceived as belonging to the same surface, thus reducing segregation. On the other hand, if the effect is related to a fundamental iso-orientation suppression, changing the nature of the gap would not affect performance.

## Conclusions

Little is known about the disruptive effect of central visual shapes on peripheral perception, because the general view is that peripheral vision is at least partly extrapolated from central vision (reviewed in Stewart et al., 2020). In this study, we demonstrated that this is not always true and that central vision may have a disruptive effect on peripheral vision. Studying the CRIP effect implies studying our percept of the visual world. The patterns used in this study represent a proxy for everyday visual scenes, which differ between the center and periphery. Our results can be further extended to investigate more complex and ecological scenes by using the same paradigm presented in this study.

## Supplementary Material

Supplement 1
