# Supplementary material for: The CRIP effect: How a pattern in central vision interferes with perception of a pattern in the periphery
Source: J Vis. 2025 Feb 26;25(2):10. doi: 10.1167/jov.25.2.10 (PMC11875039; doi:10.1167/jov.25.2.10)
Supplement: Supplement 1 [file jovi-25-2-10_s001.pdf]

Plots with single participants

Figures 1S-6S show the results from Experiments 1-5. In these plots, each participant is represented by a dot of a different colour.

Figure 1S shows the *d’* for each condition in Experiment 1.

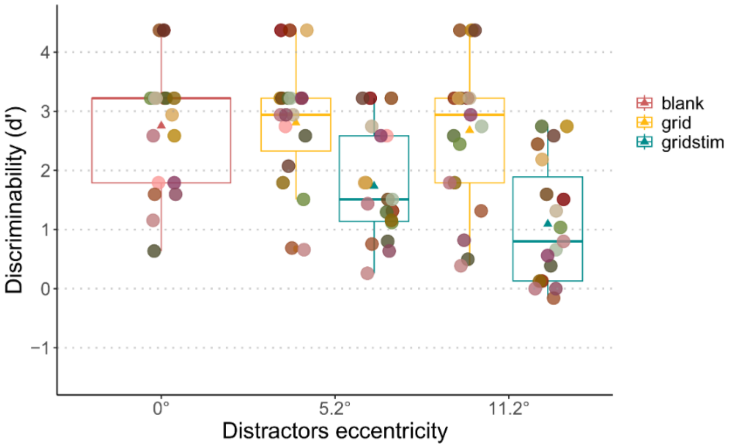

***Figure 1S* – Performance in Experiment 1.** *d’* is plotted as a function of the distractors eccentricity. The 0° eccentricity (blank in the legend) corresponds to the baseline condition, with nothing in the centre. The label ‘grid’ represents the condition in which the grid was present without distractors, while ‘gridstim’ stands for the presence of both grid and distractors. Each coloured dot represents one participant and the triangles are the averages for each condition.

Figure 2S shows the *d’* for each manipulation in Experiment 2.

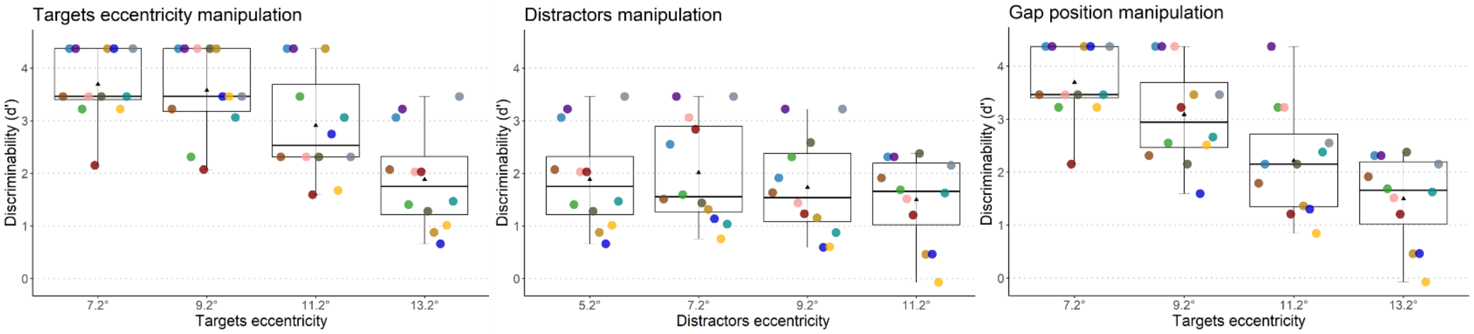

***Figure 2S* – *d’* for the three manipulations in Experiment 2**. Each plot represents one manipulation. Coloured dots represent each participant and the black triangles represent the mean. In the gap position manipulation both targets and distractors eccentricity change.

Figure 3S shows the *d’* for each manipulation in Experiment 3.

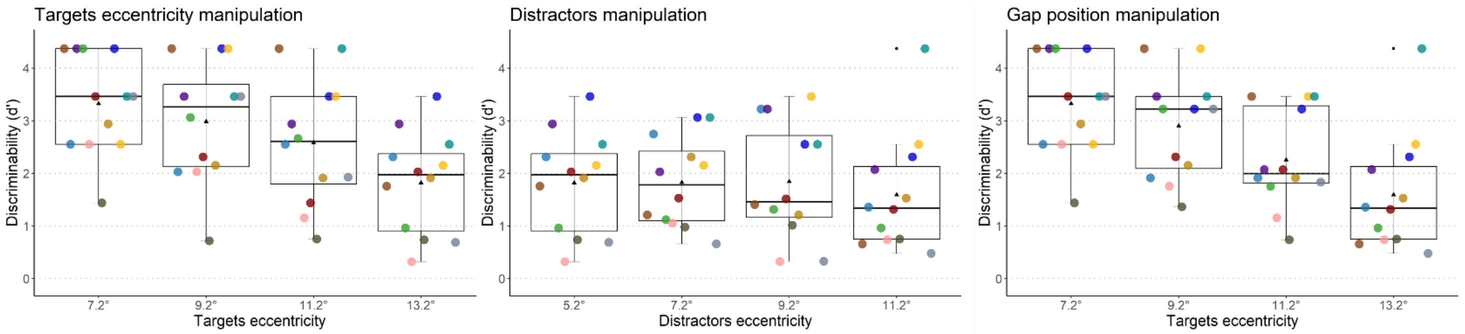

***Figure 3S* – *d’* for the three manipulations in Experiment 3**. Each plot represents one manipulation. Coloured dots represent each participant and the black triangles represent the mean. In the gap position manipulation both targets and distractors eccentricity change.

Figure 4S shows the *c* for both targets and distractors manipulation in Experiment 4.

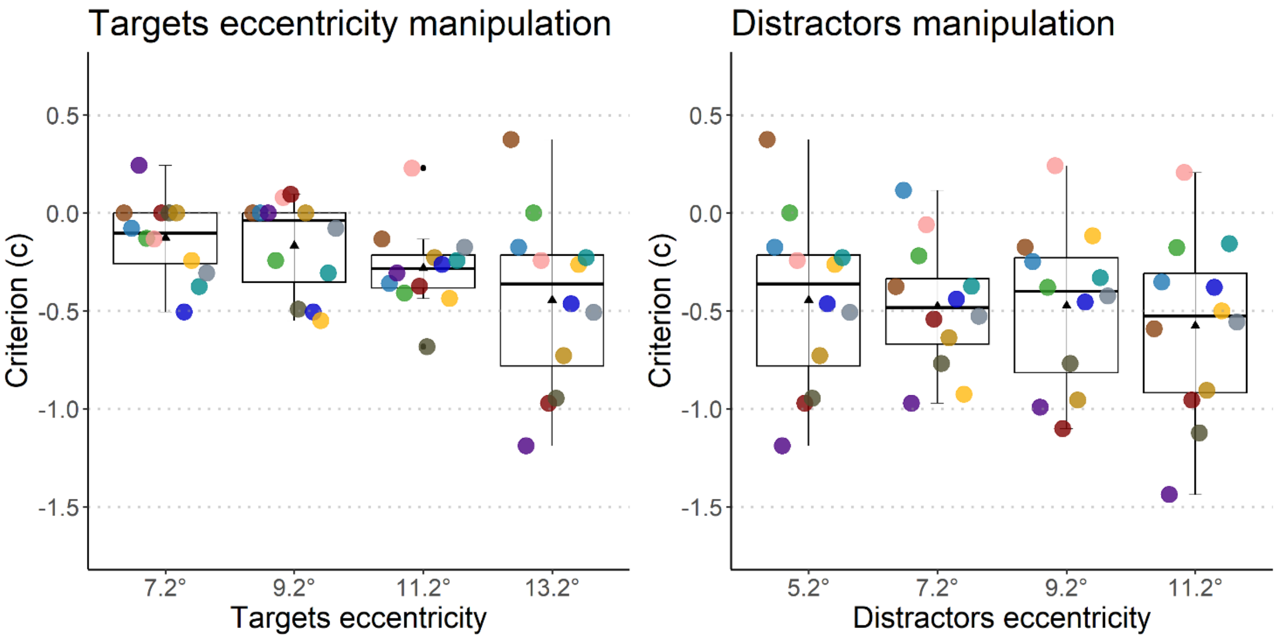

***Figure 4S* – Criterion variation for Experiment 4**. On the left panel the criterion is plotted as a function of targets eccentricity. On the right panel, it is plotted as a function of distractors eccentricity. Each coloured dot represents one participant. The black triangles represent the averages for each eccentricity.

Figure 5S shows the *d’* for each manipulation and condition in Experiment 4.

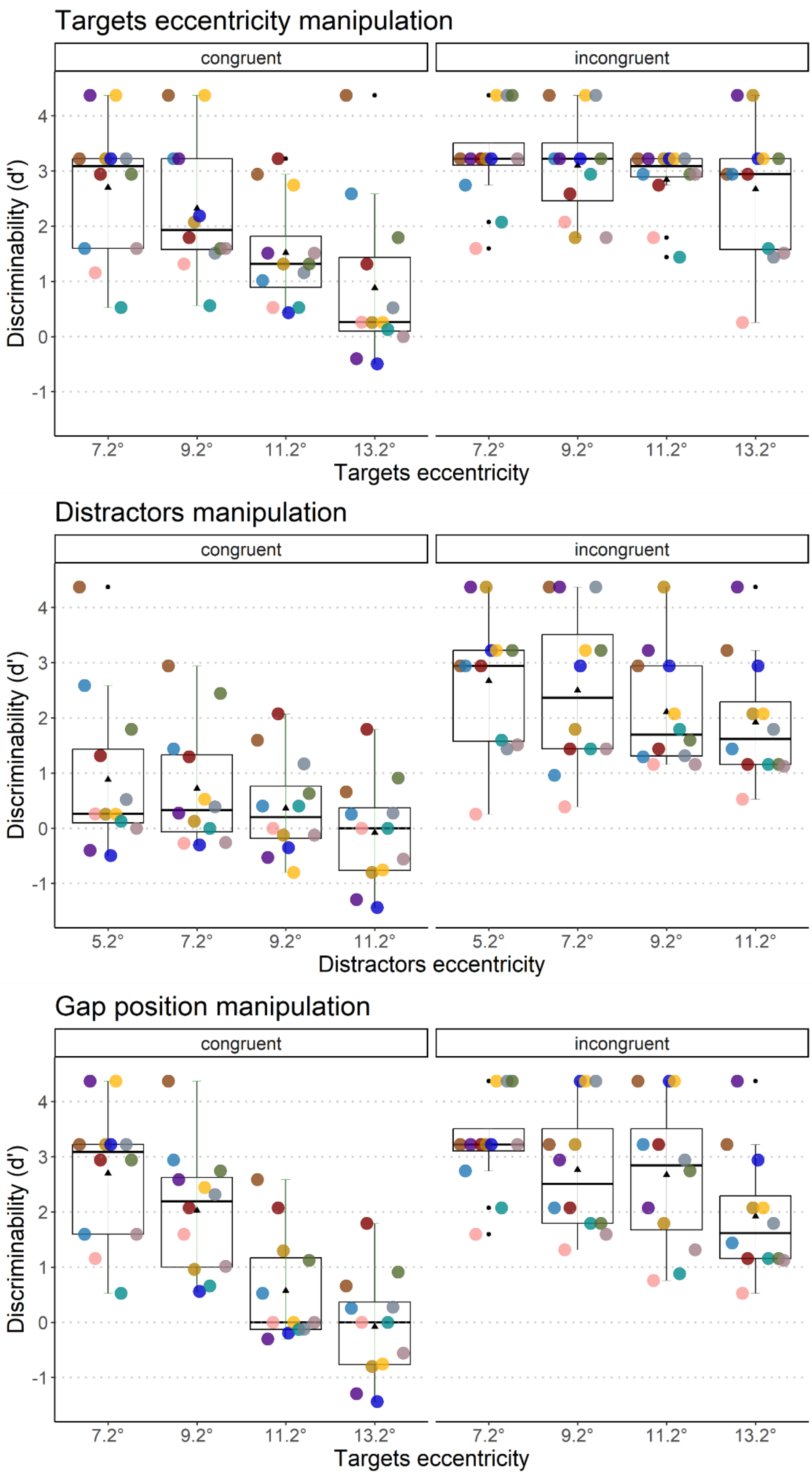

Figure 5S **– *d'* for the three manipulations in Experiment 4, divided for congruent and incongruent condition.** Each plot represents one manipulation. In the gap position manipulation both targets and distractors' eccentricity change. Each coloured dot represents one participant. The black triangles represent the averages for each eccentricity.

Figure 6S shows the results from Experiment 5 block 1 (left panel) and 2 (right panel).

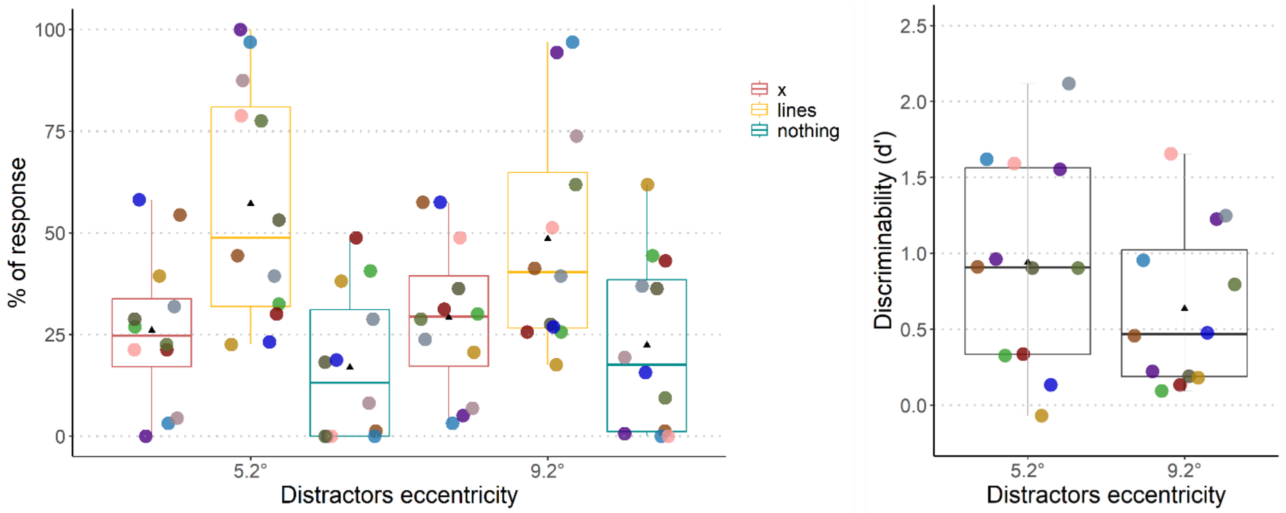

Figure 6S **– Results from block 1 (left panel) and block 2 (right panel) of Experiment 5**. The left panel shows the percentage of responses for each category (×s, lines, nothing). The responses are divided for the two distractors eccentricities. The right panel shows the *d’* for each distractors eccentricity. For both plots, each dot represents a participant, and the black triangles represent the averages.

Experiment 1: Analysing the whole sample

Figure 7S shows the *d’* for each condition of the full sample of 30 participants.

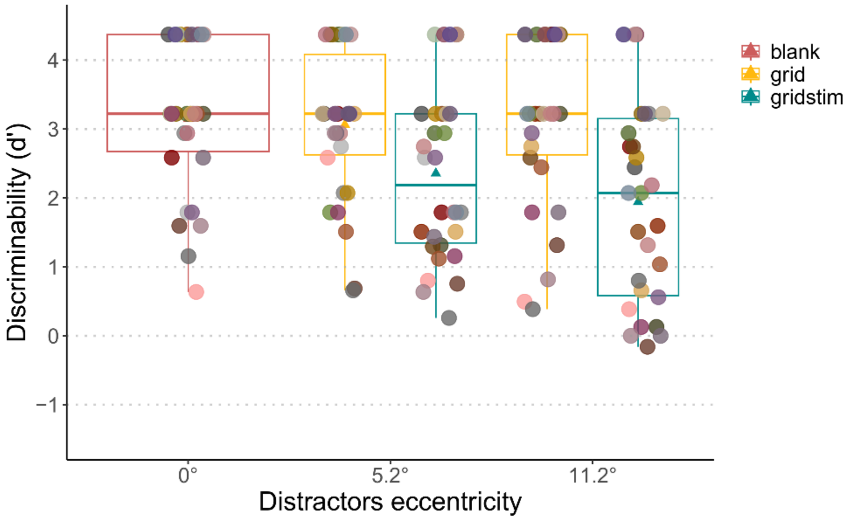

Figure 7S **– Performance in Experiment 1 for the whole sample of participants.** The *d’* is plotted as a function of the distractors eccentricity. The 0° eccentricity (blank in the legend) corresponds to the baseline condition, with nothing in the centre. The label ‘grid’ represents the condition in which the grid was present without distractors, while ‘gridstim’ stands for the presence of both grid and distractors. Each coloured dot represents one participant and the triangles are the averages for each condition.

The ANOVA on the *d’* as dependent variable, condition as independent variable and participants as the random factor revealed an effect of condition ($F(4,116) = 16.496$, $p < .001$). Post-

hoc pairwise comparisons are reported in Table S1. The post-hoc replicated the results from the main analyses, except for the comparison between the two distractors conditions, which was not significant. This may be due to the fact that the excluded participants were at ceiling in the small gap condition, thus the drop in performance was less evident when adding those participants to the sample. However, the drop was still present, as shown in Figure 7S.

***Table S1* – Post-hoc comparisons for Experiment 5.** The compared conditions are shown in the contrast column. In the contrast column, grid represents the condition without distractors, grid + stim represents the condition with both grid and distractors; the large gap corresponds to distractors eccentricity of 5.2° and small gap corresponds to 11.2°.

contrast	estimate	SE	df	t-ratio	p-value
baseline vs grid large gap	0.0825	0.195	116	0.424	0.7688
baseline vs grid small gap	-0.0381	0.195	116	-0.196	0.8452
baseline vs grid + stim large gap	0.7891	0.195	116	4.052	0.0002 *
baseline vs grid + stim small gap	1.2046	0.195	116	6.185	<.0001 *
grid large gap vs grid small gap	-0.1206	0.195	116	-0.619	0.7160
grid large gap vs grid + stim large gap	0.7067	0.195	116	3.629	0.0009 *
grid small gap vs grid large gap	1.2427	0.195	116	6.381	<.0001 *
grid + stim large gap vs grid + stim small gap	0.4155	0.195	116	2.133	0.0560

Experiment 4: additional analyses for *c* and *d’*

We report additional analyses on *c* for Experiment 4. First, we substituted in the formula from the main manuscript corrI with the rate of correct responses for the -45° orientation and errC with the rate of incorrect responses for the +45° orientation. The new formula was the following:

$$c = \frac{-z(corrLeft) + z(errRight)}{2}$$

Where corrLeft represents the rate of correct responses for the -45° orientation, and errRight represents the rate of incorrect responses for the +45° condition, that is, when targets were +45° and the participants reported -45°. In this way, it was possible to disentangle between a congruency bias and a general bias towards one or the other orientation. More specifically, a negative *c* would mean a bias towards responding -45° independently of the congruency between targets and distractors orientation. On the other hand, a positive *c* would mean a bias towards responding +45°. *A c* close to zero would mean no bias towards one or the other orientation.

The average c was 0.08, which is close to zero and implies no bias towards a specific orientation. Figure 8S shows the c variation as a function of targets-distractors distance.

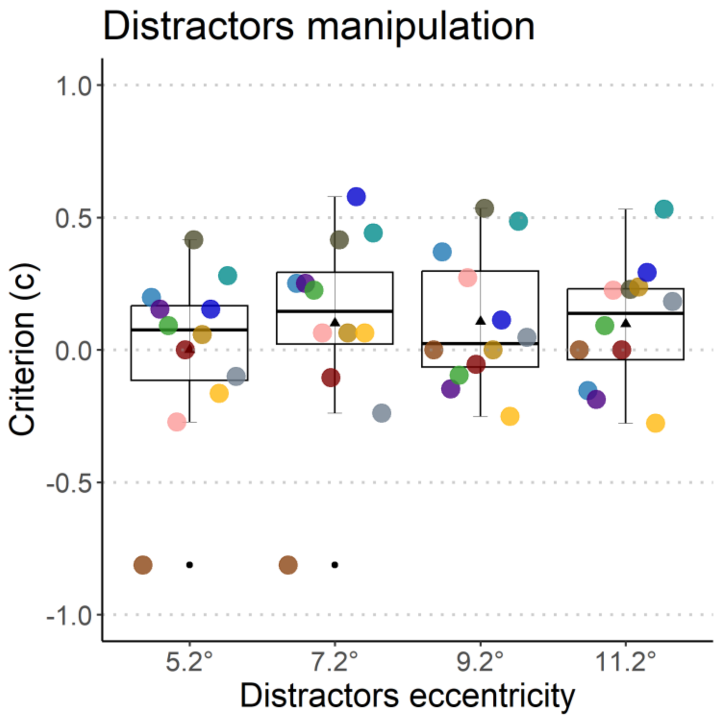

***Figure 8S* – Criterion variation.** The criterion is plotted as a function of the distractors eccentricity. Each coloured dot represents one participant. The black triangles represent the averages for each eccentricity.

The mean d' of the pooled data was 0.99. An ANOVA on the d' with distractors eccentricity as the fixed factor and participant as the random factor revealed an effect of distractors ($F(3,33) = 6.91$, $p = .001$).

We then analysed criterion from Experiment 4 diving the conditions by the orientation of the central lines (distractors).

The average c for the -45° oriented distractors was 0.76, which implies a bias towards reporting the opposite (+45°) orientation for the targets. The d' was 1.31. The average c for the +45° oriented distractors was -0.53, which implies a bias towards reporting the opposite (-45°) orientation for the targets. The d' was 1.46.

The c for the two groups confirms the incongruency bias, while the absence of a difference between the two d' confirms that the difference found when splitting the data into a congruent and incongruent group does not depend on the distractors orientation per se, but on the congruency between central and peripheral orientations.

Experiment 5: Grid position analysis

We reran the same analysis of Experiment 5 adding the grid position factor. For block 1 we compared a null model without predictors with the models that included distractors eccentricity and grid position in all possible combinations. The model selection revealed the best model to be that with only distractors eccentricity as a predictor, which had a total amount of predictive power of 100% compared to the full set of models (Table S2).

Table S2 **– Model selection for block 1 of Experiment 5.** For each model we report the Bayesian Information Criterion (BIC). The smaller the BIC, the better the fit. The models are labelled as their factors in the form of a '+' when the interaction is not included and of a '×' when the interaction is included. Null model represents the model without any factor. K is the number of parameters in the mixed model that includes fixed and random effects. Delta BIC for each model refers to the difference in the BIC score between the current model and the best model. BIC weight is the proportion of the total amount of predictive power.

**Model**	**K**	**BICc**	**Delta BIC**	**BIC weight**
Distractors eccentricity	4	7772.99	0.00	1.00
Grid position + distractors eccentricity	6	7787.21	14.22	0.00
Null model	2	7788.44	15.45	0.00
Grid position × distractors eccentricity	8	7802.55	29.56	0.00
Grid position	4	7802.68	29.69	0.00

Figure 9S shows the results from block 2 divided for grid position (on vs off). percentages for each.

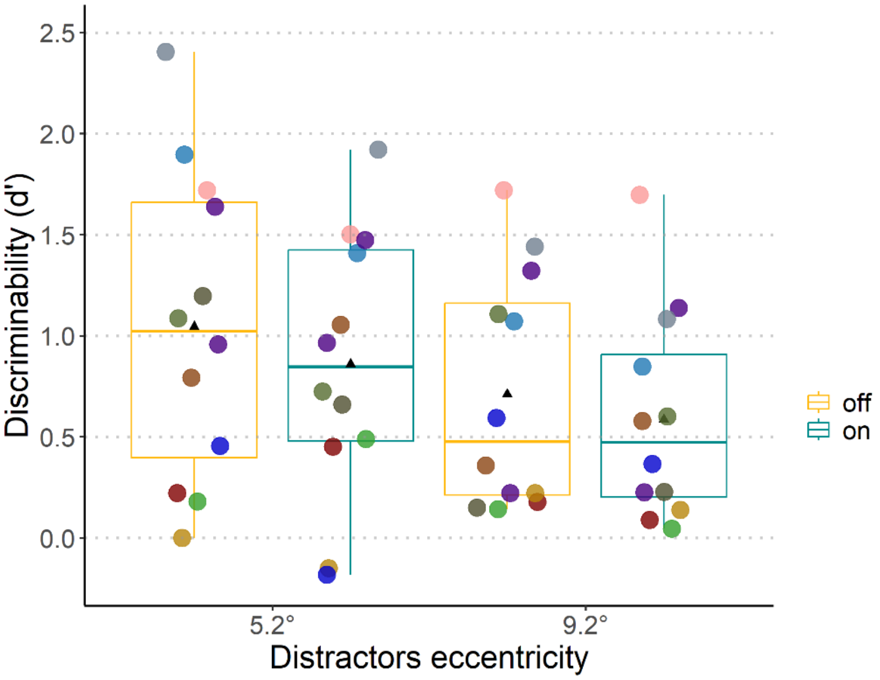

Figure 9S **– Results from block 2 of Experiment 5.** The plot shows the d' for each both distractors eccentricities and grid positions (on vs off). The black triangles represent the means. Each coloured dot represents one participant.

The ANOVA on the linear model revealed a significant effect of distractors eccentricity ($F(1,33) = 13.673$, $p < .001$), no effect of grid position ($F(1,33) = 3.551$, $p = .068$) and no interactions ($F(1,33) = .141$, $p = .710$).
